# Novel gain-tuning for sliding mode control of second-order mechanical systems: theory and experiments

**DOI:** 10.1038/s41598-023-37562-7

**Published:** 2023-06-29

**Authors:** Nguyen Xuan-Mung, Ngoc Phi Nguyen, Dinh Ba Pham, Nhu-Ngoc Dao, Huu Tiep Nguyen, Thanh Ha Le Nhu Ngoc, Mai The Vu, Sung Kyung Hong

**Affiliations:** 1 Faculty of Mechanical and Aerospace Engineering, Sejong University, Seoul, 05006 South Korea; 2 Department Mechanical Engineering, Vietnam Maritime University, Haiphong, 180000 Vietnam; 3 Department of Computer Science and Engineering, Sejong University, Seoul, 05006 South Korea; 4 Department of Quantum and Nuclear Engineering, Sejong University, Seoul, 05006 South Korea; 5 Department of Mechatronics Engineering, Ho Chi Minh City University of Technology and Education, Ho Chi Minh City, 700000 Vietnam; 6 School of Intelligent Mechatronics Engineering, Sejong University, Seoul, 05006 South Korea; 7 Department of Convergence Engineering for Intelligent Drone, Sejong University, Seoul, 05006 Korea

**Keywords:** Aerospace engineering, Electrical and electronic engineering, Mechanical engineering

## Abstract

The sliding mode control is well-known as a useful control technique that can be applied in several real-world applications. However, a straightforward and efficient process of selecting the sliding mode control gains remains a challenging but interesting topic. This paper investigates a novel gain tuning method for the sliding mode control of second-order mechanical systems. Firstly, we obtain relations between the gains and the natural and damping ratio of the closed-loop system. Secondly, the time constant of the system’s actuators and the system response performance criteria, including settling time and delay time, are taken into consideration to determine appropriate ranges of the gains. These gain ranges allow control designers to select the controller gains in a time-saving manner and ensure that the desired system performance is met and the actuators work properly. Finally, the proposed method is applied to the gain tuning process of a sliding mode altitude controller for an actual quadcopter unmanned aerial vehicle. Simulation and experimental results demonstrate the applicability and effectiveness of this method.

## Introduction

Sliding mode control (SMC) is a well-known robust control technique for complex nonlinear systems under parametric uncertainties and external disturbances^1,2^. The studies about SMC have been pursued in broad areas that investigate the different aspects of this control strategy. The topics of this type of research range from the first-order SMC^3,4^, high-order SMC^5^, and discrete-time SMC^6^ to terminal SMC^7^ as well as event-triggered SMC^8^. With several advantages in order to delivery robust and stable performance to a wide class of systems, the SMC technique has been implemented as an automatic controller for numerous applications in the fields of robotics^9^, industrial automation^10,11^, power electronics^12^, automotive^13^, autonomous ground vehicles^14^, and aerospace^15–17^.

Among the research areas mentioned above, the first-order SMC has emerged because it is simple to design and implement, requires low computational resources, and delivers satisfactorily rapid and robust performance. The first-order SMC was first introduced decades ago, but this approach has continuously attracted the special attention from scientists worldwide. An event-triggered SMC for delta operator systems was investigated by^18^. Based on a first-order SMC and published in the same year, which is 2020, the work presented by^19^ proposed an SMC for general uncertain systems, while one by^20^ introduced an event-triggered SMC for high-order systems. By looking at more specific real-world applications, the first-order SMC is seen in many studies in a variety of sectors. Qi et al.^21^ took advantage of an SMC to increase the DC voltage gain and decrease the voltage stress on the power switch in a DC-DC power boost converter. An SMC was also used to solve the fault tolerance^22^, attitude^23^, and precision landing^24^ control problems of aerospace systems. The presence of SMC is also seen in the study of pressure regulation of an oxygen mask in an oxygen supply system^25^. Another work in ocean engineering, presented by^26^, utilized a first-order SMC to develop a motion compensation base that cranes and drilling platforms can be placed on to eliminate the effect of wave-induced ship motions. Further, the SMC’s application is also found in biomedical science, where it is used as a guide for non-pharmaceutical intervention to control the COVID-19 pandemic^27^. In addition, there are a number of other first-order-SMC-based works that can be easily found in the literature^28–32^.

The above observations show that second-order mechanical systems actuated by actuators are omnipresent and play essential roles in many real-world applications. And the SMC technique is applied widely to control such systems. However, in most related existing studies^18–20,33–35^, the SMC was designed and tuned independently of the actuators’ dynamics. Therefore, the actuators could be over-operated, and the saturation phenomenon could occur. Dealing with such problems requires additional efforts in control design and system analysis, which are usually complicated. Besides, in the works presented by^18–20,33–35^, the SMC’s gain selection methods only guaranteed the systems’ stability, while the desired performance (such as settling time and delay time) could only be achieved after tuning the gains by time-consuming trials and errors.

Motivated by the above observations, this paper focuses on finding out a method for determining the SMC’s gains with a mathematical, systematic, and straightforward tuning process. Our work contributes to state-of-the-art knowledge in three main ways:

1. Unlike the works in^18–20,33–35^, our novel method establishes a gain range, which allows control designers to pick appropriate gains quickly. The selected gains not only guarantee the system’s stability but also ensure the system’s desired performance (including settling time and delay time) is satisfied simultaneously. Hence, we can achieve satisfactory system performance without wasting time tuning the gains through trials and errors.
2. Also, unlike the exsiting studies^18–20,33–35^, our work takes the system’s actuator dynamics into consideration to ensure the actuators are not over-operated. Therefore, from practical and economic points of view, the proposed method enhances the system’s operation quality and saves costs as it lenthens the actuators’ lifespan.
3. Through experiments with an actual quadcopter UAV, the theory presented in this paper is demonstrated as being highly reliable and applicable. It is worth noting that quadcopters are one typical class of second-order systems. Hence, the successful implementation of the proposed method to this class indicates that it can also work for a broad range of systems in many real-world applications.

The remainder of this paper is organized as follows: The preliminaries and the problem statement are presented in Section “Preliminaries and problem statement”, the proposed method in Section “Main results”, an illustrative example with simulation and experimental results and discussion in Section “Illustrative example”, and conclusions in Section “Conclusions”.

## Preliminaries and problem statement

### Performance specification of feedback control systems

**Figure 1:**
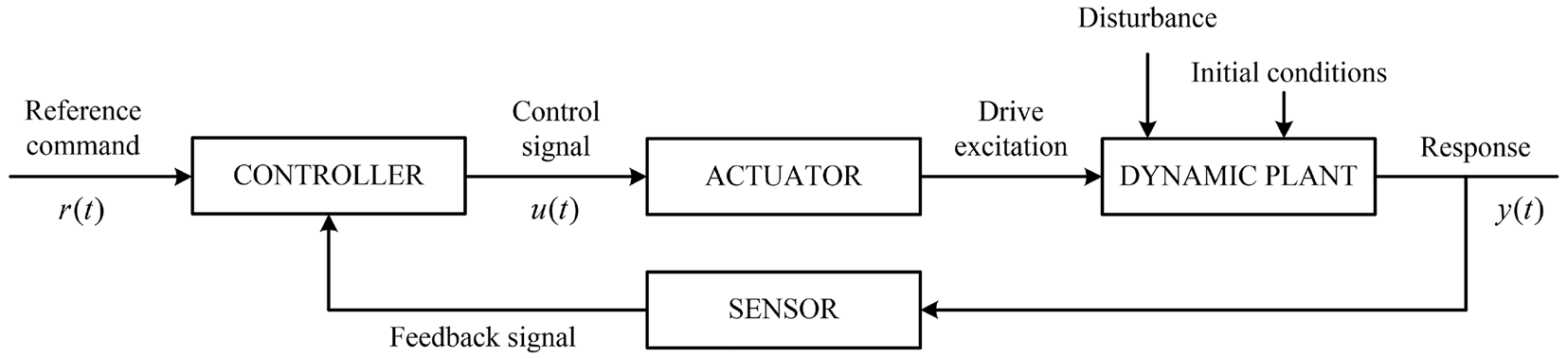
General configuration of a feedback control system^36^.

The operation and evaluation of a control system (Fig. 1) are based on a set of performance specifications that typically include speed of response, stability, and accuracy^37^. In particular, for a first-order system, whose dynamic model is expressed as follows (with *k* being a dc gain):

1 $$\begin{aligned} \varvec{\tau } \dot{\textbf{y}}(t) + \textbf{y}(t) = k\textbf{u}(t) \end{aligned}$$

the time constant matrix, $\varvec{\tau } = \text {diag}(\tau _1, \tau _2, ..., \tau _n)$, is used as a specification in the system’s performance evaluation. Meanwhile, for a second-order system, several parameters can be used. A simple model for a second-order system can be described as

2 $$\begin{aligned} \ddot{\textbf{y}}(t) + 2\varvec{\zeta } \varvec{\omega }_n \dot{\textbf{y}}(t) + \varvec{\omega }_n^2 \textbf{y}(t)= \varvec{\omega }_n^2 \textbf{u}(t) \end{aligned}$$

where, $\textbf{y}(t) \in \mathbb {R}^{n}$ is the system’s state vector, $\varvec{\zeta } = \text {diag}(\zeta _1, \zeta _2, ..., \zeta _n)$ and $\varvec{\omega }_n = \text {diag}(\omega _1, \omega _2, ..., \omega _n)$ are the matrices of damping ratio and natural frequency, respectively, of the system. The delay time, $\varvec{\tau }_d$, and settling time (2%), $\varvec{\tau }_s$, of this system are calculated as

3 $$\begin{aligned} \varvec{\tau }_d = ({\textbf{I}_n+0.7\varvec{\zeta }}){\varvec{\omega }_n^{-1}} \end{aligned}$$

and

4 $$\begin{aligned} \varvec{\tau }_s = {4}({\varvec{\zeta }\varvec{\omega }_n})^{-1} \end{aligned}$$

where, $\textbf{I}_n$ is the identity matrix in $\mathbb {R}^{n \times n}$.

The system’s actuators play essential roles in satisfying predefined performance requirements. In particular, the system’s response, which can be fast or slow or accurate or inaccurate, depends on the response speed and the accuracy of the actuators. When it comes to the response speed, the actuators’ time constant is considered. For the system to have any change in its motions, it always takes a time interval longer than the actuators’ time constant.

### Sliding mode control

Consider the class of systems

5 $$\begin{aligned} {\left\{ \begin{array}{ll} { \dot{\textbf{x}}_1 = \textbf{x}_2}\\ \dot{\textbf{x}}_2 = \textbf{f}(\textbf{x}) + \textbf{h}(\textbf{x})\textbf{u} \end{array}\right. } \end{aligned}$$

where $\textbf{x} = [\textbf{x}_1$ $\textbf{x}_2]^T$ is the state ($\textbf{x}_1, \textbf{x}_2 \in \mathbb {R}^{n}$); $u \in \mathbb {R}^m, (m \le n)$ the control input; $\textbf{f}$ and $\textbf{h}$ are sufficiently smooth functions with $\text {rank}(\textbf{h}(\textbf{x})) = m$.

To design the sliding mode controller for this system, the sliding surface^4^ is firstly designed as:

6 $$\begin{aligned} \varvec{\sigma } = \textbf{x}_2 - \phi (\textbf{x}_1) \end{aligned}$$

where the function $\phi (\textbf{x}_1) \in \mathbb {R}^{n}$ is chosen such that when the motion is restricted to the surface, and the model

7 $$\begin{aligned} \dot{\textbf{x}}_1 = \phi (\textbf{x}_1) \end{aligned}$$

is asymptotically stabilized at the origin. Next, to bring $\varvec{\sigma }$ to zero in finite time and have it maintained there for all future time, the control input $\textbf{u}$ is designed as

8 $$\begin{aligned} \textbf{u} = {\mathbf {h_{inv}}(\textbf{x})}[-\textbf{f}(\textbf{x}) + {{\partial \phi (\textbf{x}_1)}\over {\partial \textbf{x}_1}}\dot{\textbf{x}}_1 - \varvec{\gamma }(\textbf{x})\text {sat}(\varvec{\sigma })] \end{aligned}$$

where $\mathbf {h_{inv}}(\textbf{x})$ denotes the Moore-Penrose inverse^38^ of $\textbf{h}(\textbf{x})$, $\varvec{\gamma }(\textbf{x}) \ge 0$ is a continuous function, and sat($\varvec{\sigma }$) is the saturation function, which is defined as $\text {sat}(\varvec{\alpha }) = [\text {sat}(\alpha _1), \text {sat}(\alpha _2), ..., \text {sat}(\alpha _n)]^T$, where, $\alpha _i$ $(i=1,2,...,n)$ is the element of vector $\varvec{\alpha } \in \mathbb {R}^n$, and

9 $$\begin{aligned} { \text {sat}(\alpha _i) = {\left\{ \begin{array}{ll} 1, \quad \alpha _i \ge 1 \\ \alpha _i, \quad -1< \alpha _i < 1 \\ -1,\quad \alpha _i\le -1 \end{array}\right. }} \end{aligned}$$

In the existing studies and many practical applications^15,28^, it is seen that the functions $\varvec{\phi }(\textbf{x}_1)$ and $\varvec{\gamma }(\textbf{x})$ are chosen as

10 $$\begin{aligned} \varvec{\phi }(\textbf{x}_1) = -\textbf{K}_1 \textbf{x}_1 \end{aligned}$$

and

11 $$\begin{aligned} \varvec{\gamma }(\textbf{x}) = \textbf{K}_2 \end{aligned}$$

with $\textbf{K}_1$ and $\textbf{K}_2 \in \mathbb {R}^{n \times n}$ being positive definite diagonal matrices to be chosen.

#### Remark 1

From a practical point of view, it is common that a mechanical system has its closed-loop system’s time constant much larger than its actuators’ time constants. Therefore, for the sake of simplicity, the actuator dynamics is ignored in control design procedures in most existing studies^18–20,33–35^. Hence, it is seen in (8) that the control law $\textbf{u}$ is designed without considering the system’s actuator dynamics. However, in this work, the actuator dynamics will be taken into consideration to obtain appropriate values for the controller’s gains $\textbf{K}_1$ and $\textbf{K}_2$.

## Main results

### Relationship between the SMC gains and the closed-loop system’s performance specifications

This subsection presents a method of determining the SMC’s gains from the expected natural frequency and damping ratio of the closed-loop second-order system.

#### Theorem 1

If the controller (8) is applied to the system (5) then the following holds:

12 $$\begin{aligned} {{\left\{ \begin{array}{ll} \textbf{K}_1 + \textbf{K}_2 = 2\varvec{\zeta } \varvec{\omega }_n\\ \textbf{K}_2 \textbf{K}_1 = \omega ^2_n \end{array}\right. }} \end{aligned}$$

with $\varvec{\omega }_n$ and $\varvec{\zeta } \in \mathbb {R}^{n \times n}$ being the expected diagonal matrices of natural frequency and damping ratio, respectively, of the closed-loop system.

#### Proof

From (6), (8), with $\phi (\textbf{x}_1)$ and $\varvec{\gamma }(\textbf{x})$ being chosen as in (10) and (11), we have

13 $$\begin{aligned} {\begin{aligned} \dot{\varvec{\sigma }}&= \dot{\textbf{x}}_2 + \textbf{K}_1 \dot{\textbf{x}}_1 = \textbf{f}(\textbf{x}) + \textbf{h}(\textbf{x}) \textbf{u} + \textbf{K}_1 \dot{\textbf{x}}_1 \\&= -\textbf{K}_2 \text {sat}(\varvec{\sigma }) \end{aligned}} \end{aligned}$$

Consider the following Lyapunov function candidate: $V = {1 \over 2} \varvec{\sigma }^T \varvec{\sigma }$. Then, the time derivative of *V* is $\dot{V} = \varvec{\sigma }^T \dot{\varvec{\sigma }} = -\varvec{\sigma }^T \textbf{K}_2 \text {sat}(\varvec{\sigma })$.

Let $D_{\sigma } = \{\sigma _i: |\sigma _i| < 1\}$ and $D^{\text {sat}}_{\sigma } = \{\sigma _i: |\sigma _i| \ge 1 \}$. Let $N_{sat}$ be the size of $D^{\text {sat}}_{\sigma }$. Thus, $0 \le N_{sat} \le n$. The size $N_{sat}$ can fall into one of the following cases.

*Case 1*. If $$N_{sat} > 0$$.

Thus, we have

14 $$\begin{aligned} {\begin{aligned} \dot{V}&= -\sum _{\sigma _i \in D_\sigma }^{}{k_{2i}\sigma _i^2} - \sum _{\sigma _j \in D^{\text {sat}}_{\sigma }}^{}{k_{2j}|\sigma _j|} \quad < 0, \quad \forall \varvec{\sigma } \ne 0 \end{aligned}} \end{aligned}$$

where, $k_{2i} (i=1,2,...,n)$ is the diagonal element of $\textbf{K}_2$.

The observation in (14) indicates that if any element $\sigma _j$ of the sliding surface, $\varvec{\sigma }$, lies in the range $|\sigma _j| \ge 1$, it will be forced back to the range $|\sigma _j| < 1$ and asymptotically approaches the origin as long as the controller gains, $\textbf{K}_1$ and $\textbf{K}_2$, are positive definite. The above analysis leads to examining the following case, Case 2.

*Case 2*. If $N_{sat} = 0$, then $\text {sat}(\varvec{\sigma }) = \varvec{\sigma }$.

We have,

15 $$\begin{aligned} {\begin{aligned} \dot{V}&= -\sum _{\sigma _i \in D_\sigma }^{}{k_{2i}\sigma _i^2} \quad < 0, \quad \forall \varvec{\sigma } \ne 0 \end{aligned}} \end{aligned}$$

The control input $\textbf{u}$ can be rewritten as

16 $$\begin{aligned} {\begin{aligned} \textbf{u}&= {\mathbf {h_{inv}}(\textbf{x})}[-\textbf{f}(\textbf{x}) - \textbf{K}_1 \dot{\textbf{x}}_1 - \textbf{K}_2 \varvec{\sigma }] \\&= {\mathbf {h_{inv}}(\textbf{x})}[-\textbf{f}(\textbf{x}) - \textbf{K}_1 \dot{\textbf{x}}_1 - \textbf{K}_2(\textbf{x}_2 + \textbf{K}_1 \textbf{x}_1)] \end{aligned}} \end{aligned}$$

From (5) we have

17 $$\begin{aligned} {\begin{aligned} \ddot{\textbf{x}}_1&= \dot{\textbf{x}}_2 = \textbf{f}(\textbf{x}) + \textbf{h}(\textbf{x})\textbf{u} \end{aligned}} \end{aligned}$$

Substituting (16) into (17) yields

18 $$\begin{aligned} {\begin{aligned} \ddot{\textbf{x}}_1&= -\textbf{K}_1\dot{\textbf{x}}_1 - \textbf{K}_2(\textbf{x}_2 + \textbf{K}_1 \textbf{x}_1) \\&= - \textbf{K}_1\dot{\textbf{x}}_1 - \textbf{K}_2 \textbf{K}_1 \textbf{x}_1 - \textbf{K}_2 \textbf{x}_2 \end{aligned}} \end{aligned}$$

Manipulating (18), we have

19 $$\begin{aligned} {\begin{aligned} \ddot{\textbf{x}}_1&= -\textbf{K}_1 \dot{\textbf{x}}_1 - \textbf{K}_2 \textbf{K}_1 \textbf{x}_1 - \textbf{K}_2 \dot{\textbf{x}}_1 \\&= - (\textbf{K}_1 + \textbf{K}_2) \dot{\textbf{x}}_1 - \textbf{K}_2 \textbf{K}_1 \textbf{x}_1 \end{aligned}} \end{aligned}$$

It is seen in (19) that the dynamics of $\textbf{x}_1$ now is in the form of a second-order system in (2)

20 $$\begin{aligned} \ddot{\textbf{x}}_1 + 2\varvec{\zeta } \varvec{\omega }_n \dot{\textbf{x}}_1 + \omega ^2_n \textbf{x}_1 = 0 \end{aligned}$$

where,

21 $$\begin{aligned} {{\left\{ \begin{array}{ll} \textbf{K}_1 + \textbf{K}_2 = 2\varvec{\zeta } \varvec{\omega }_n \\ \textbf{K}_2 \textbf{K}_1 = \omega ^2_n \end{array}\right. }} \end{aligned}$$

This completes the proof of Theorem 1. $\square$

### Appropriate gain ranges

Since the system’s control performance criteria may vary with its applications, the set of controller’s gains of a system used for one task can be significantly different from the ones that are used for another task. Therefore, in many cases, the gains obtained by solving (21) work but may not satisfy some control performance requirements, and a gain-tuning is needed. In this subsection, we discuss the limits the gains can reach during the tuning process.

#### Theorem 2

For a given actuator time constant, $\varvec{\tau }_A := \text {diag}(\tau _{a1}, \tau _{a2}, ..., \tau _{an})$, the closed-loop system which consists of the system (5) and the controller (8) will be stable and meet the set of performance criteria including the desired settling time (2%), $\varvec{\tau }_s := \text {diag}(\tau _{s1}, \tau _{s2}, ..., \tau _{sn})$, if the following holds:

1. The diagonal element $k_{1i}$ $(i=1,...,n)$, of $\textbf{K}_1$ satisfies:
  $$\begin{aligned} {\left\{ \begin{array}{ll} { {1 \over {\varvec{\tau }_{si}}} \le k_{1i} \le {1 \over {\varvec{\tau }_{ai}}} } \qquad \qquad \qquad \qquad \qquad \qquad \qquad \qquad (22) \\ { k_{1i} < {8 \over {\varvec{\tau }_{si}}} }\qquad \qquad \qquad \qquad \qquad \,\,\,\,\qquad \qquad \qquad \qquad (23)\\ {rank(\textbf{K}_1) = n} \qquad \qquad \qquad \qquad \qquad \qquad \qquad \,\,\,\qquad (24) \end{array}\right. } \end{aligned}$$
2. The diagonal element $k_{2i}$ $(i=1,...,n)$, of $\textbf{K}_2$ satisfies:
  $$\begin{aligned} {\left\{ \begin{array}{ll} { k_{2i} \le 7k_{1i} } \qquad \qquad \qquad \qquad \qquad \qquad \qquad \qquad \qquad \qquad \qquad \qquad (25)\\ { {1 \over {\sqrt{k_{2i} k_{1i}}}}+{{0.35(k_{1i} + k_{2i})} \over {k_{2i} k_{1i}}} \ge \tau _{ai} } \qquad \qquad \qquad \qquad \qquad \qquad \qquad \qquad (26) \end{array}\right. } \end{aligned}$$

#### Proof

Let us consider the sliding surface in (6) with $\phi (\textbf{x}_1)$ being replaced by $-\textbf{K}_1 \textbf{x}_1$. Thus, we have

27 $$\begin{aligned} { \varvec{\sigma } = \textbf{x}_2 + \textbf{K}_1 \textbf{x}_1 } \end{aligned}$$

From (5) and (27), we have:

28 $$\begin{aligned} { \dot{\textbf{x}}_1 + \textbf{K}_1 \textbf{x}_1 = \varvec{\sigma } } \end{aligned}$$

Consider (28) as a first-order system, we have its time constant, $\varvec{\tau }_1 := \text {diag}(\tau _{11}, \tau _{12}, ..., \tau _{1n})$, calculated as

29 $$\begin{aligned} {\varvec{\tau }_1 = \textbf{K}_1^{-1} } \end{aligned}$$

It is obvious that

30 $$\begin{aligned} { \varvec{\tau }_{ai} \ll \varvec{\tau }_{1i} \le \varvec{\tau }_{si} } \end{aligned}$$

From (29) and (30), one can obtain

31 $$\begin{aligned} { {1 \over {\varvec{\tau }_{si}}} \le k_{1i} \le {1 \over {\varvec{\tau }_{ai}}} } \end{aligned}$$

Consider the settling time (2%) parameter, $\varvec{\tau }_2 := \text {diag}(\tau _{21}, \tau _{22}, ..., \tau _{2n})$, which is calculated as in (4), of the second-order system in (20):

32 $$\begin{aligned} \varvec{\tau }_2 = {{4}({\varvec{\zeta }\varvec{\omega }_n})^{-1}} \end{aligned}$$

From (12) and (32), we have

33 $$\begin{aligned} { \varvec{\tau }_2 = 8(\textbf{K}_1 + \textbf{K}_2)^{-1} } \end{aligned}$$

Since it is desired that $\varvec{\tau }_2 = \varvec{\tau }_s$, (33) can be rewritten as

34 $$\begin{aligned} { \textbf{K}_2 = 8{\varvec{\tau }_s}^{-1} - \textbf{K}_1 } \end{aligned}$$

Because $k_{2i} > 0$, we have

35 $$\begin{aligned} { k_{1i} < {8 \over \varvec{\tau }_{si}} } \end{aligned}$$

The equations (31) and (35) complete the proof of (22) and (23).

We now moving on proving (25) and (26). Obviously, we have:

36 $$\begin{aligned} { \varvec{\tau }_{2i} \ge \varvec{\tau }_{1i} } \end{aligned}$$

From (29), (33), and (36), we have

37 $$\begin{aligned} { {1 \over k_{1i}} < {8 \over {k_{1i} + k_{2i}}} } \end{aligned}$$

or

38 $$\begin{aligned} {k_{2i} \le 7 k_{1i} } \end{aligned}$$

We also take the delay time, $\varvec{\tau }_3 := \text {diag}(\tau _{31}, \tau _{32}, ..., \tau _{3n})$, which is calculated as in (3), of the system (20) into consideration:

39 $$\begin{aligned} { \varvec{\tau }_3 = ( \textbf{I}_n + 0.7\varvec{\zeta }){\varvec{\omega }_n}^{-1} } \end{aligned}$$

In order for the actuators to not be over-operated, the expected delay time should be larger than $\varvec{\tau }_A$. Thus, we have

40 $$\begin{aligned} { \tau _{3i} \ge \tau _{ai} } \end{aligned}$$

Substituting (39) into (40) yields

41 $$\begin{aligned} { {{1 + 0.7 \zeta _i} \over {\varvec{\omega }_{ni}}} \ge \tau _{ai} } \end{aligned}$$

From (12) in Theorem 1, we have

42 $$\begin{aligned} {{\left\{ \begin{array}{ll} {1 \over \omega _{ni}} = {1 \over \sqrt{k_{2i} k_{1i}}} \\ {\zeta _i \over \omega _{ni}} = {{k_{1i} + k_{2i}} \over {2 k_{2i} k_{1i}}} \end{array}\right. }} \end{aligned}$$

Substituting (42) into (41) yields

43 $$\begin{aligned} {{1 \over {\sqrt{k_{2i} k_{1i}}}}+{{0.35(k_{1i} + k_{2i})} \over {k_{2i} k_{1i}}} \ge \tau _{ai} } \end{aligned}$$

With $\textbf{K}_1$ chosen as in (22) and (23), the appropriate values of $\textbf{K}_2$, which satisfy both (38) and (43) can be obtained. This completes the proof of (25) and (26). $\square$

#### Remark 2

**Figure 2:**
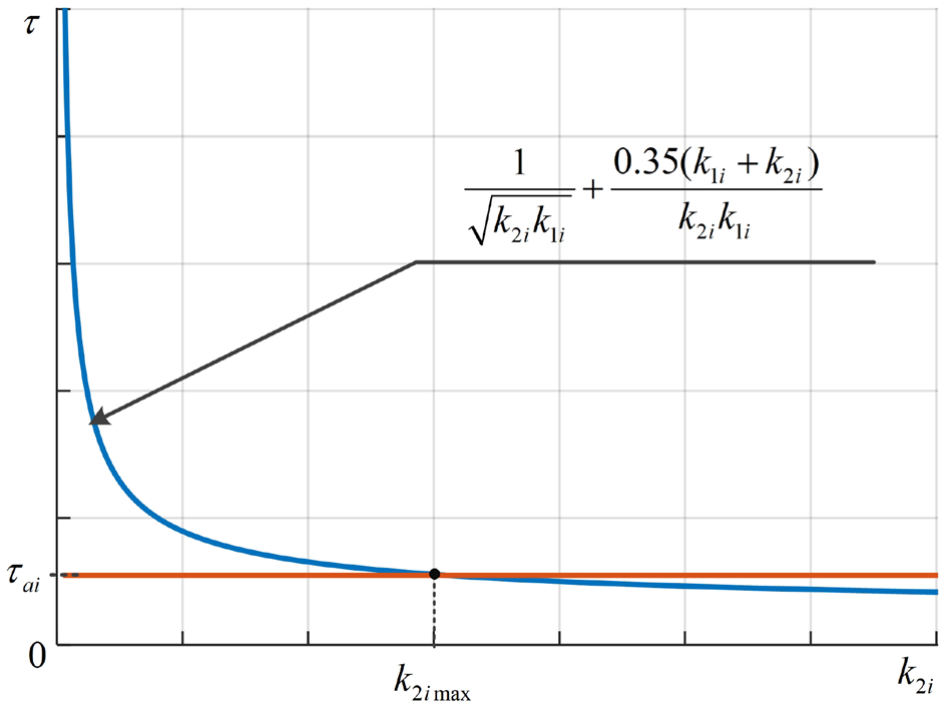
The maximum candidate value ($k_{2i\text {max}}$) of the element $k_{2i}$ of $\textbf{K}_2$ can be determined through a graphical method.

By using equation (26) in Theorem 2, a graphical method, which is illustrated in Fig. 2, can be used to determine the maximum candidate value of $k_{2i}$.

#### Remark 3

It is worth noting that not only the delay time but also the rise time and peak time are the parameters that represent the speed of response of the system (5). These parameters can also be used to determine the controller gains. However, we used the delay time in this paper because it is helpful to obtain simple formulas like (25) and (26). Besides, since the settling time is related to the stability level of the system, we consider it alongside the delay time to achieve the most appropriate controller gains, which ensure both the response speed and the stability degree of the system.

#### Remark 4

The actuator time constant, $\varvec{\tau }_A$, is a measure of the motor’s speed reaction time upon change in the terminal voltage (or the control input, in other words). Meanwhile, is defined as time constant of the system in (28), which means the reaction time of the system’s response upon the changes in the sliding surface (or the control input, in other words). Therefore, $\varvec{\tau }_A$ directly affects $\varvec{\tau }_1$ and the system’s settling time, $\varvec{\tau }_2$ (the higher the $\varvec{\tau }_A$, the larger the $\varvec{\tau }_1$ and $\varvec{\tau }_2$). Hence, by considering $\varvec{\tau }_A$, our gain selection rules in Theorem 2 ensure the system’s desired performance is satisfied without over-operating the actuators.

## Illustrative example

The design and gain-tuning process of the SMC applied to the quadcopter UAV system is presented in this section to illustrate the applicability and effectiveness of the proposed method.

### Quadcopter platform

#### Hardware and software

**Figure 3:**
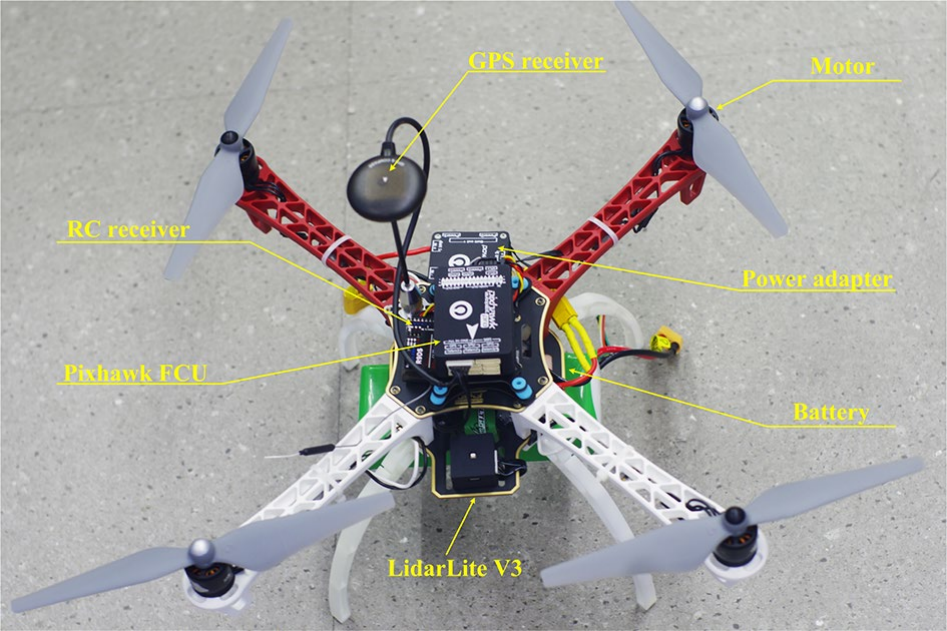
The experimental quadcopter platform used for the experiment in this study.

**Figure 4:**
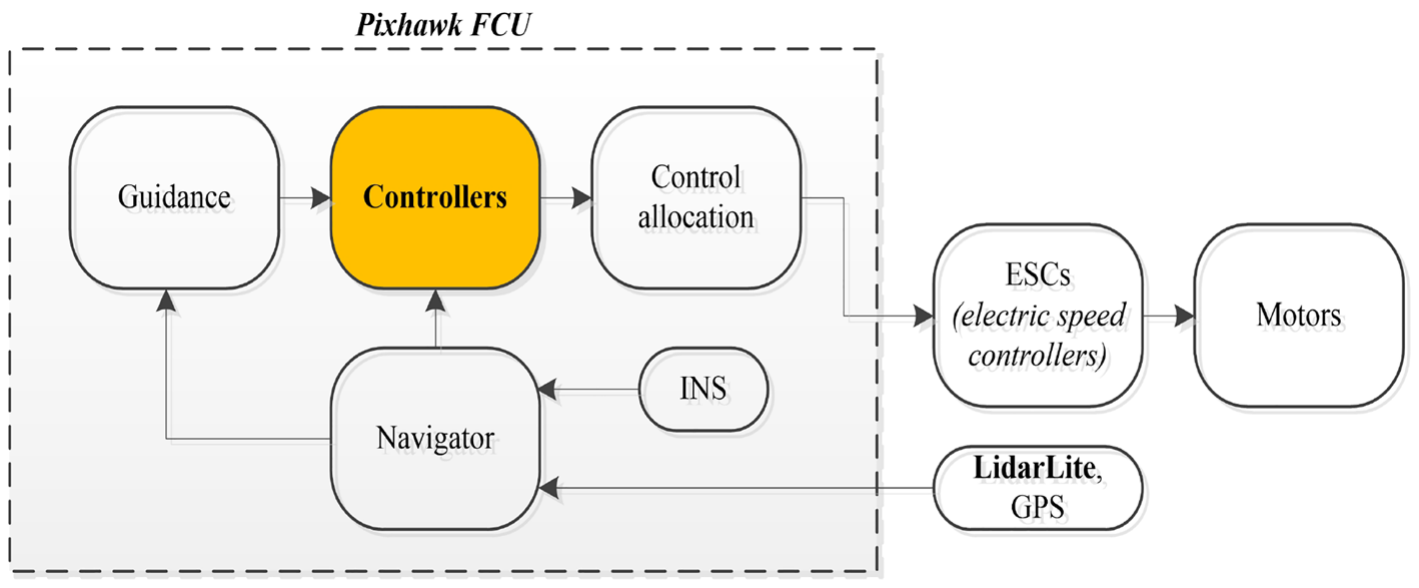
The system signal flow diagram.

We used a quadcopter as the experimental platform (Fig. 3), which is operated by an onboard flight computer unit (FCU) Pixhawk. The quadcopter attitude and acceleration are provided by an inertial navigation system (INS). We used a commercially available laser ranging sensor LidarLite V3 to measure the altitude and a commercial GPS receiver module to determine the vehicle’s position. The quadrotor’s translational velocities are extracted from an INS/GPS/Lidar Lite sensor fusion through an extended Kalman filter. Besides, a power supplying system (including a battery and a power adapter module), a set of remote control transmitter/receiver for the manual pilot, and a set of radio telemetry transmitter/receiver for the ground station monitoring were used. The motors of the quadcopter have a time constant of 0.1 s. The the vehicle attitude controller is operated at a frequency of 400 Hz, and the SMC altitude controller runs at 100 Hz. The block diagram in Fig. 4 briefly describes the signal flows in the experimental system.

#### Dynamics model

Since the quadcopter’s dynamics was introduced and verified in several existing studies^39,40^, we only describe it briefly here. Four motors of the quadcopter generate four thrust forces $F_i$ ($i=$1,...,4) that have a relation with four control inputs ($u_i$) as

44 $$\begin{aligned} {\left\{ \begin{array}{ll} u_1 = F_1 + F_2 + F_3 + F_4 \\ u_2 = l(F_2 - F_4) \\ u_3 = l(F_3 - F_1) \\ u_4 = c_{fm}(-F_1 + F_2 - F_3 + F_4) \end{array}\right. } \end{aligned}$$

The full cascaded dynamics model of the quadcopter is well-known as

45 $$\begin{aligned} {\left\{ \begin{array}{ll} \ddot{z} = -g + \frac{1}{m}(\text {cos}\phi \text {cos}\theta )u_1 \\ \ddot{\phi } = \frac{I_y-I_z}{I_x}\dot{\theta }\dot{\psi } + \frac{1}{I_x}u_2 \\ \ddot{\theta } = \frac{I_z-I_x}{I_y}\dot{\psi }\dot{\phi } + \frac{1}{I_y}u_3 \\ \ddot{\psi } = \frac{I_x-I_y}{I_z}\dot{\phi }\dot{\theta } + \frac{1}{I_z}u_4 \end{array}\right. } \end{aligned}$$

where, *l* is the quadcopter’s arm length; $c_{fm}$ the force-to-momentum coefficient; $I_x, I_y, I_z$ the inertia momentum; *m* the mass; *g* the gravitational acceleration. *x*, *y* denote the position; *z* the altitude; and $\phi , \theta , \psi$ the attitude of the vehicle in the inertial frame {*E*}. Details of the quadcopter’s dynamical parameters are listed in Table 1.

**Table 1:** The quadcopter’s dynamical parameters.

Symbol	Value	Unit
*m*	1.8	kg
$I_x, I_y, I_z$	0.013, 0.012, 0.021	kg $\hbox {m}^2$
*l*	0.225	m
*g*	9.81	m/$\hbox {s}^2$
$c_{fm}$	0.02	m
$\varvec{\tau }_A$	0.1	s

The method described in Sections “Preliminaries and problem statement” and “Main results” is applied in order to design a sliding mode altitude tracking controller for the quadcopter, and to tune the controller’s gains.

Let us define the tracking error as

46 $$\begin{aligned} e_z = z_d - z \end{aligned}$$

where, $z_d$ is the desired altitude. Thus, the first-order and second-order derivatives of $e_z$ can be calculated as

47 $$\begin{aligned} \dot{e}_z = \dot{z}_d - \dot{z} \end{aligned}$$

and

48 $$\begin{aligned} \ddot{e}_z = \ddot{z}_d + g - \frac{1}{m}(\text {cos}\phi \text {cos}\theta )u_1 \end{aligned}$$

Let ${x}_1 = e_z$ and ${x}_2 = \dot{e}_z$, we have

49 $$\begin{aligned} {\left\{ \begin{array}{ll} \dot{{x}}_1 = {x}_2 \\ \dot{{x}}_2 = \ddot{z}_d + g - \frac{1}{m}(\text {cos}\phi \text {cos}\theta )u_1 \end{array}\right. } \end{aligned}$$

We can see that (49) has the form of (5). Hence, we can apply the method presented in Section “Main results” to design a sliding mode altitude tracking controller for a quadcopter and tune the controller’s gains.

### Quadcopter’s sliding mode altitude tracking controller

A sliding surface is introduced as

50 $$\begin{aligned} {\sigma } = \dot{e}_z + {k}_1 e_z \end{aligned}$$

with ${k}_1$ being a positive number to be chosen. Then, following Section “Main results”, the control law is obtained as

51 $$\begin{aligned} u_1 = {m \over {cos\phi cos\theta }}[\ddot{z}_d + g + {k}_1 \dot{e}_z + {k}_2 {\text {sat}({\sigma })] } \end{aligned}$$

where, ${k}_2$ is a positive gain to be decided.

Our goal now is to choose the appropriate values for ${k}_1$ and ${k}_2$ such that the controller (51) satisfies the control criteria described in Table 2 and exhibits safe altitude tracking performance.

**Table 2:** The quadcopter’s altitude tracking control criteria.

Parameter	Value	Unit
Desired delay time	$>\varvec{\tau }_A$	s
Desired settling time	4	s

### Simulation results

To examine the impact of the controller’s gains on the system’s performance, and verify the effectiveness of our method through this, we conducted a simulation with several scenarios, which are described as below.

(1) First, the appropriate gains are chosen and applied to the quadcopter system following the method presented in Section “Main results”. After that, the controller gains are set with values that are (2) close to the appropriate gain range’s boundary, and (3) beyond the appropriate gain range’s boundary.

#### Appropriate gains for the most satisfactory performance

**Figure 5:**
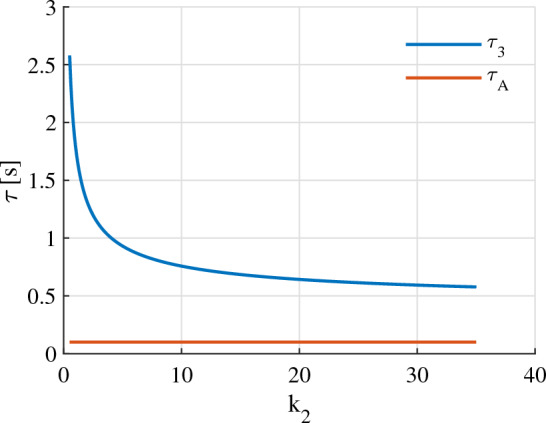
The motor constant ($\varvec{\tau }_A$) and the delay time ($\varvec{\tau }_3$) numerically calculated when ${k}_1 = 0.9$.
- Check the system’s performance and fine-tune the gains.
  We are going to check the system’s performance and slightly tune the above-obtained gains to achieve the best performance. It is seen in Fig. 6 that the altitude controller works stably, and the altitude tracking performance is roughly satisfactory. However, as shown in the inset of this figure, the settling time (2%) is about 4.7 s and does not satisfy the predefined condition, i.e., ${\tau }_2 = 4$ s, even though it is not far from the desired value. Therefore, we continue tuning the gains for the controller to meet the control criteria. A slight increase of ${k}_1$ is followed by an update of ${k}_2$, which is ruled by (25) and (26). After a few times of fine-tuning, it turns out that we achieved the most satisfactory performance when ${k}_1 = 1.05$ and ${k}_2 = 7$ (Fig. 7).

The gains ${k}_1$ and ${k}_2$ are chosen as followings:

- Choose. $${k}_1$$
  Following Eqs. (22) and (23) in Theorem 2, with the motor time constant ${\tau }_A = 0.1$ seconds and the desired settling time ${\tau }_s = 4$ seconds, we have
  52 $$\begin{aligned} 0.25 \le {k}_1 < 2.0 \end{aligned}$$
  Let us give ${k}_1$ the middle value of the above range, i.e., ${k}_1 = 0.9$.
- Choose. $${k}_2$$
  With $0.25 \le {k}_1 < 2.0$, the inequality (25) yields
  53 $$\begin{aligned} {k}_2 \le 6.3 \end{aligned}$$
  An appropriate value of ${k}_2$ also needs to satisfy (26) which is graphically described in Fig. 5. It is seen in Fig. 5 that the (26) holds for all ${k}_2$ lie in the range from 0 up to 6.3. Therefore, let us choose ${k}_2 = 6.2$.

**Figure 6:**
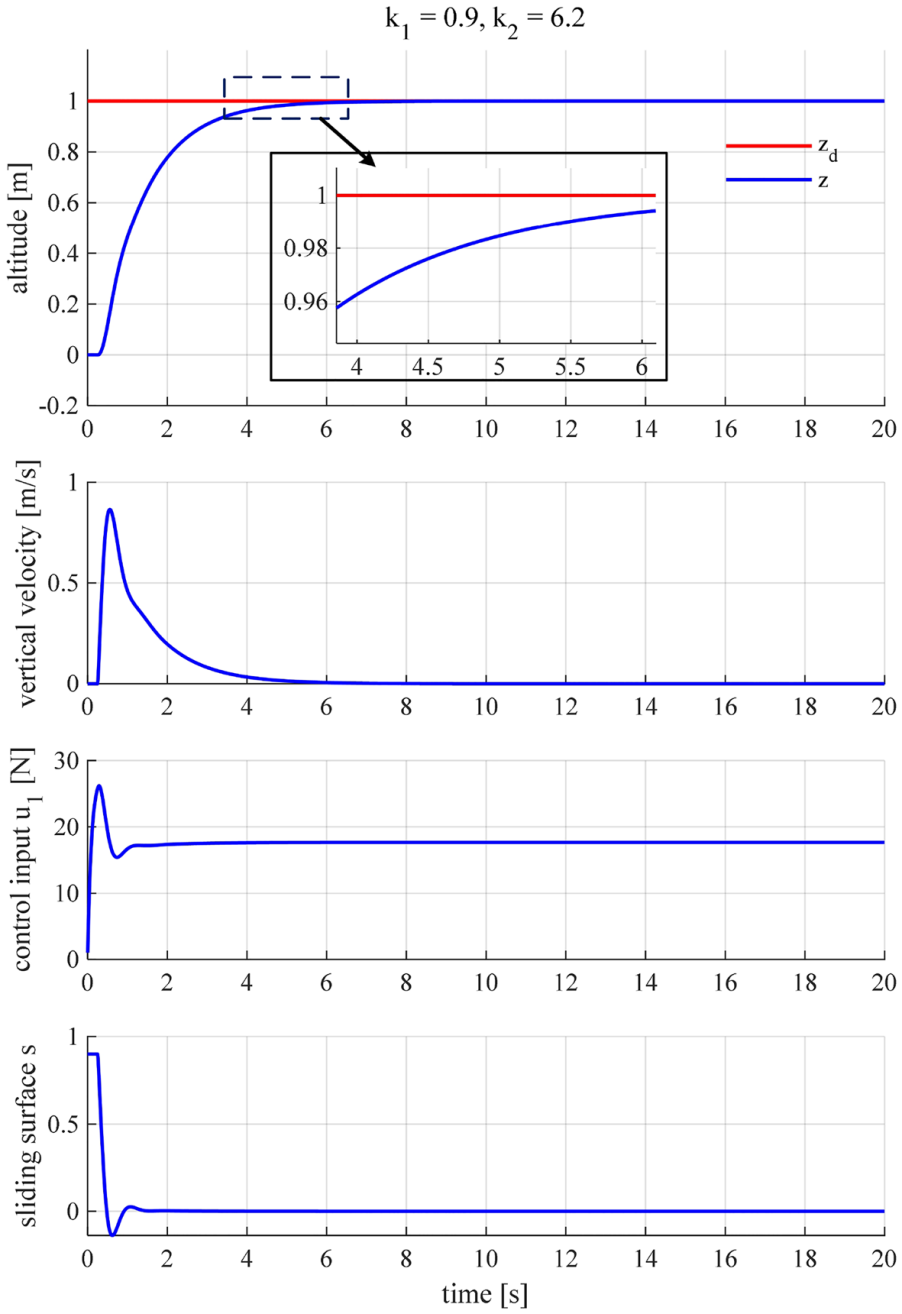
The quadcopter’s altitude performance when ${k}_1 = 0.9$ and ${k}_2 = 6.2$.

**Figure 7:**
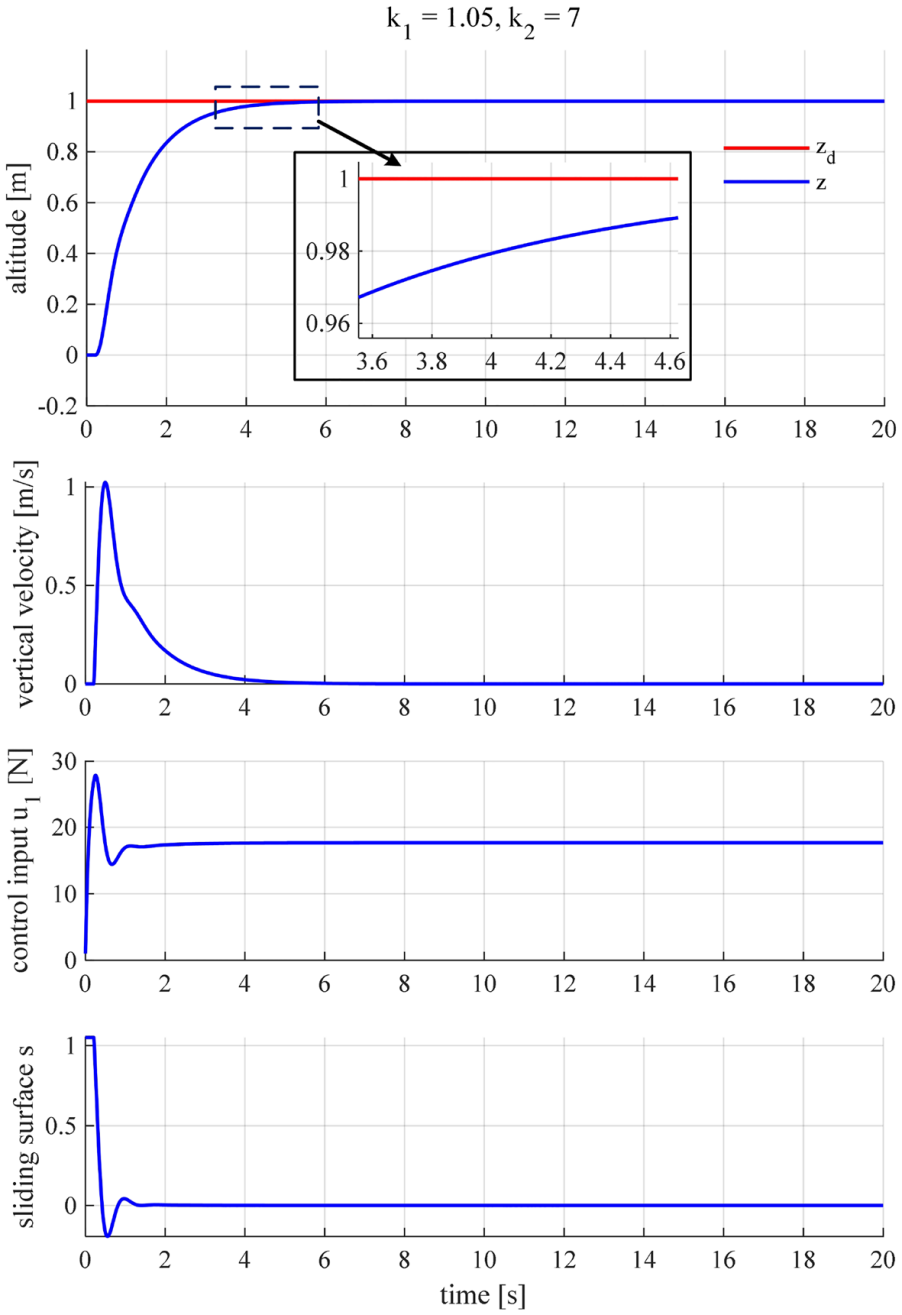
The SMC altitude controller exhibits the most satisfactory performance when ${k}_1 = 1.05$ and ${k}_2 = 7$.

.

#### Gains close to the appropriate gain range’s boundary

**Figure 8:**
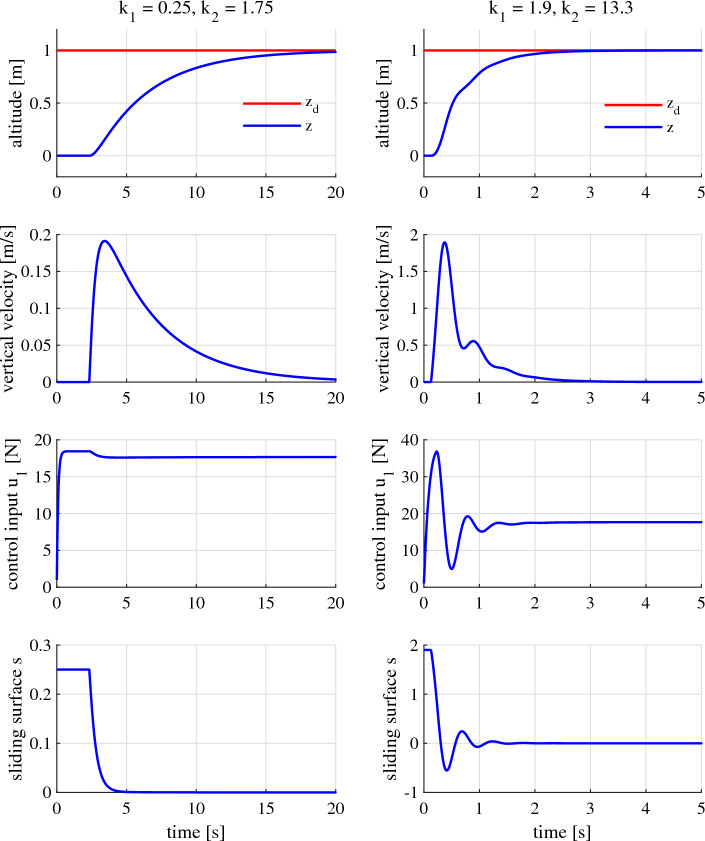
Performance of the altitude, vertical velocity, control input, and sliding surface when ${k}_1$ and ${k}_2$ are set closed to the appropriate gain range’s boundary.

Let us now examine the system’s performance when the gains are set at the values close to the appropriate gain range, which is determined in the previous Section “Appropriate gains for the most satisfactory performance“. That is, the gain ${k}_1$ is going to be set as 0.25 and 1.9 (Fig. 8).

With ${k}_1 = 0.25$, following (25), we have ${k}_2 \le 1.75$. By choosing ${k}_2 = 1.75$, we have the system exhibits too weak response to the command. The maximum vertical speed only reaches 0.19 m/s, and the system takes about 19.5 seconds to achieve the 2% settling state as a result.

On the other hand, when we set ${k}_1 = 1.9$ and choose ${k}_2 = 13.3$ (since ${k}_2 \le 13.3$), the system response becomes markedly faster. It can be seen (in Fig. 8) that the maximum vertical speed reaches 1.9 m/s and that there are some slight jerks in the quadcopter’s movement. In addition, the settling time is only 2 s in this case.

Generally, it can be said that, even though the quadcopter does not exhibit performance as good as it is desired, its flights are still stable and safe when the gains are set inside but closed to the boundary of the appropriate gain range.

#### Gains beyond the appropriate gain range

In this sub-sub section, we intentionally choose the gains ${k}_1$ and ${k}_2$, which do not satisfy (23) and (26). That is, the following is going to be selected:

54 $$\begin{aligned} {k}_1 > 10.0 \end{aligned}$$

**Figure 9:**
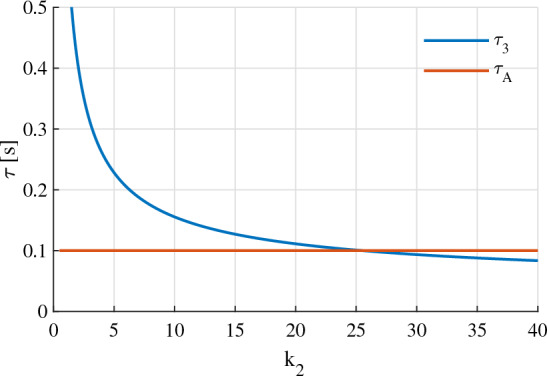
The motor constant (${\tau }_A$) and the delay time (${\tau }_3$) numerically calculated when ${k}_1 = 12$.

Let us choose ${k}_1 = 12$. With this value of ${k}_1$, we can choose ${k}_2 = 30$ so that (26) is not satisfied (Fig. 9).

**Figure 10:**
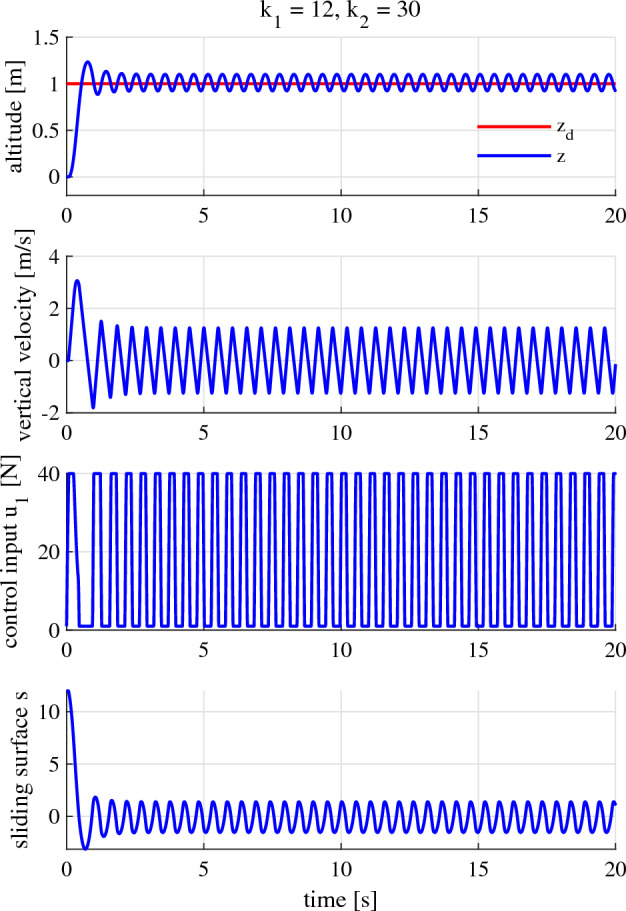
Performance of the altitude, verical velocity, control input, and sliding surface when ${k}_1 = 12$ and ${k}_2 = 30$.

The system’s performance corresponding to these values (Fig. 10) is devastating. We can see from Figs. 8 and 10 that it is difficult and time-consuming to have satisfactory performance when we choose controller gains through trials and errors because the gains we choose can easily lie beyond the appropriate range.

### Experimental results

**Figure 11:**
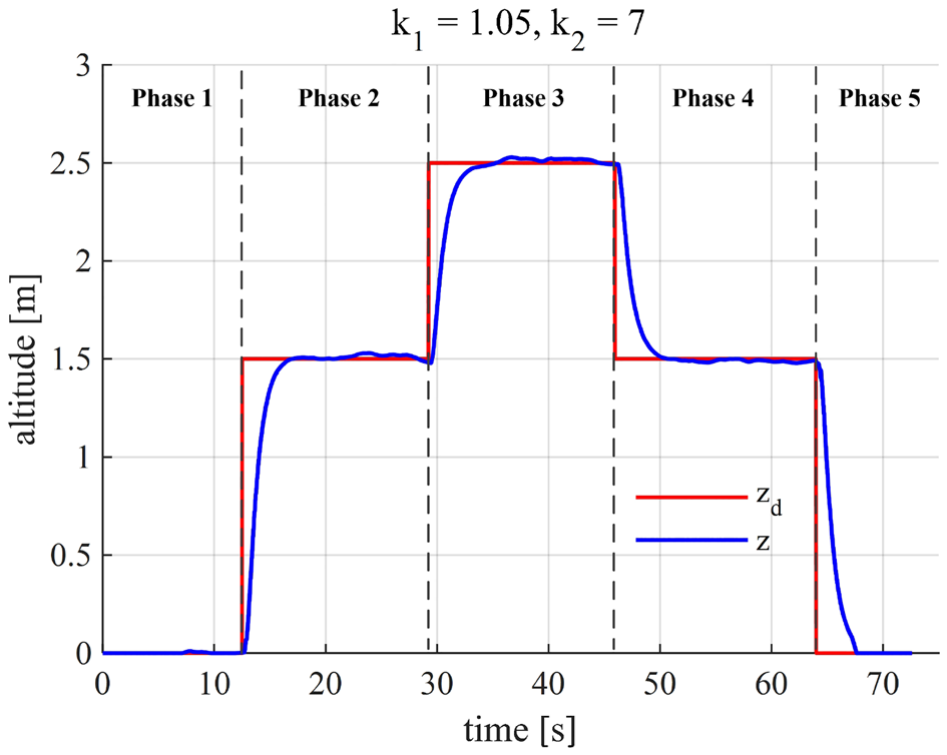
Experimental altitude performance of the quadcopter when ${k}_1 = 1.05$ and ${k}_2 = 7$.

**Figure 12:**
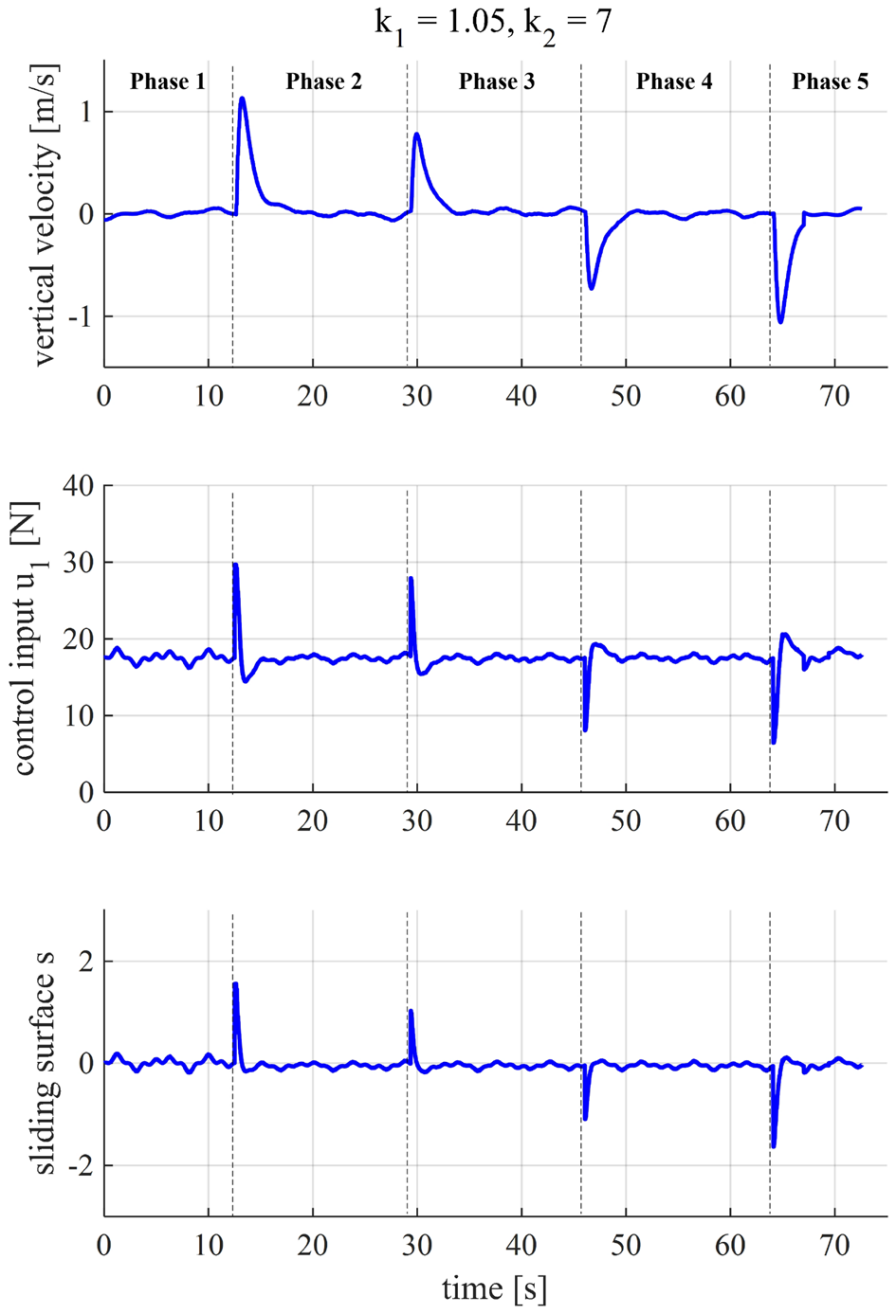
Experimental vertical velocity, control input, and sliding surface performance of the quadcopter when ${k}_1 = 1.05$ and ${k}_2 = 7$.

In this section, we demonstrate the effectiveness of the proposed method by applying it to choose the controller gains of the experimental quadcopter platform and evaluating the flight performance. The flight is conducted outdoors under actual flight conditions. The flight scenario consists of five phases, which include (1) initialization, (2) take-off, (3) ascent, (4) descent, and (5) landing (Figs. 11 and 12).

In Phase 1 (initialization), the quadcopter is turned on and armed on the ground. Afterward, when the time $t \approx 12.5$ s, Phase 2 (take-off) is enabled, the quadcopter is commanded to take-off and climb to reach an altitude of 1.5 m. At $t \approx 29.2$ s, while the quadcopter is hovering at 1.5m-height, an altitude step setpoint of 2.5 m is sent to the quadcopter that starts Phase 3 (ascent). In Phase 4 ($t \approx 46$ s), the quadcopter exhibits a descent flight from the altitude of 2.5 to 1.5 m. The final phase, starting at $t \approx 64$ s, is the landing phase in which the vehicle descent to reach the ground before being disarmed and turned off. Not only the altitdue tracking (Fig.11)but also the vertical velocity, control input, and sliding surface (Fig. 12) demonstrate the closed-loop system’s performance, thereby indicating the efficacy our controller gain-tuning process.

As per Table 3, the settling time (2%) performance of the quadcopter in each phase is slightly different yet close to the expected value, i.e., 4 s. These minor differences may be caused by several factors, such as system uncertainties, ground effect, and external disturbances. It is also seen from Table 3 that the controller gains we obtained in the previous section, without additional gain-tuning, deliver stable and safe experimental performance throughout the phases of the flight.

**Table 3:** The experimental quadcopter’s performance specifications.

Phase	Altitude setpoint (m)	Settling time (2%) (s)	Peak velocity (m/s)	Peak $u_1$ (N)
1-Init.	-	–	–	-
2-Takeoff	1.5	3.7	1.14	29.5
3-Ascent	2.5	4.4	0.78	27.9
4-Descent	1.5	3.9	− 0.75	8.1
5-Landing	0	3.6	− 1.06	6.5

## Conclusions

An SMC gain-tuning method is presented and validated in this paper. This method considers system’s actuator dynamics to ensure the actuators are not over-operated and to avoid the saturated phenomenon, which shortens the system’s lifespan and degrades its operation quality. Further, the proposed gain selection rules allow control designers to select appropriate gains for their controllers in a straightforward and time saving way. The numerical simulation and the experiment results demonstrated that the gains obtained by our method deliver stable and satisfactory performance to the system. Hence, this work can be applied to a wide range of systems that use the SMC technique. Our future study is directed to an efficient gain-tuning procedure for second-order SMC controllers based on this paper.

## Data Availability

The data that support the findings of this study are available from Guidance, Navigation, and Control Laboratory but restrictions apply to the availability of these data, which were used under license for the current study, and so are not publicly available. Data are however available from the authors upon reasonable request and with permission of Guidance, Navigation, and Control Laboratory.
